# Advances in E-Health and Mobile Health Monitoring

**DOI:** 10.3390/s22228621

**Published:** 2022-11-08

**Authors:** Pari Delir Haghighi, Frada Burstein

**Affiliations:** Department of Human-Centred Computing, Faculty of Information Technology, Monash University, Melbourne 3800, Australia

## 1. Introduction

E-health as a new industrial phenomenon and a field of research integrates medical informatics, public health and healthcare business, aiming to facilitate the provision of more accessible healthcare services, such as remote health monitoring, reducing healthcare costs and enhancing patient experience. There has been a wide array of new technologies introduced for developing mobile solutions in healthcare. Mobile apps have been the most popular trend due to the wide availability and affordable price of smartphones. The majority of smartphone-based studies employ wearable sensors and AI (artificial intelligence) techniques to collect and analyze multiple vital signs on phones and the present useful reports or recommendations to users. While e-health and mobile health monitoring are rapidly growing fields of research and practice, there still remain challenges that need to be addressed and areas that could benefit from further improvements. Despite the large number of studies on mobile health-monitoring apps, their adoption in clinical practice and studies has been limited. The lessons learned from existing studies could significantly contribute to a better understanding of how the new approaches and technologies could be implemented and used more effectively and efficiently.

This Special Issue aims to address this gap in the research by discussing and sharing the results and implications of new methods and platforms introduced in e-health and mobile health monitoring, and providing a comprehensive review and analysis of the key areas within this domain.

Eight high-quality articles were selected and published in this Special Issue. Four articles are review articles, whereas one article conducts a comparative analysis of the data-mining techniques and applies them to a real-world data set reporting on the opportunities of such approaches to improve healthcare processes. The four remaining articles present original research that introduces new methodologies, frameworks and algorithms for enhancing mobile health monitoring.

## 2. Overview of Contribution

Smartphones have been the key enabler of e-health solutions. Smartphones, at present, have built-in sensors, such as a camera and accelerometer that allow for the collection and analysis of data on the phone, using machine- and deep-learning techniques. The first article [1], by Joachim and colleagues, uses the smartphone’s camera to capture the food’s image and automatically detects the food item using AI-driven image analytics. The results are used for nutrition management and behavior intervention through the nudge theory. Kaur et al., the authors of the second article [2], introduce a smartphone-based ecological momentary assessment (EMA) method for collecting physical-activity data automatically and continuously on the phone for those with low-back pain. The collected data were then analyzed to recognize physical activities, such as sitting and walking, and understand their relationships with pain intensity. The results contribute to enhancing the data collection process in clinical trials of low-back pain that generally use self-reporting questionnaires to collect data at sparse intervals. The third article [3], by Kim et al., made technical and experimental contributions to improve the accuracy of recognizing physical activities on mobile devices. They proposed a novel algorithm that enhances transformer-based models by using the conformer model that is usually applied in speech recognition.

Smartphones and their built-in sensors provide many opportunities for health monitoring. In their article [4], Kulkarni et al. conducted a systematic review for understanding capabilities and limitations of smartphone sensing. The review presents interesting findings, including opportunities for using standardized sensing approaches and machine-learning advancements, and the predominance of mental health studies.

Mental health conditions have recently been recognized as a serious issue worldwide. A large number of studies strive to build mobile interventions to assist individuals with the monitoring and self-management of these conditions. Conversational agents and chatbots have been a promising technology in building mobile mental health apps. The success of these apps mostly relies on their AI capabilities and personalization. The fifth article [5], by Rathnayaka et al., explored the use of the behavioral activation (BA) therapy in building more effective chatbots for supporting mental health. The study’s methodology and its participatory evaluation in a pilot study setting present useful insights to the research community in this field.

Data mining has become widely used for healthcare data analytics in hospital settings. The application of such techniques requires a combination of the technical expertise of computer scientists, as well as domain knowledge to prepare the data sets for analysis and the interpretation of the results. In the sixth article [6], Gurazada, with her colleagues, explored a common problem of a prolonged length of stay in an emergency department. The article presents a review of potential data-mining techniques that have been applied to predict what factors affect the length of stay of patients. As the result, they chose an approach that was suitable for a data set provided by the hospital. The authors presented the lessons learned and identified some future research opportunities based on this application. 

E-health has experienced a remarkable evolution with regard to telehealth adoption since the start of the COVID-19 pandemic. The seventh article [7] presents a systematic review conducted by Murphy et al. that investigates the social, psychological, health and economic impacts of COVID-19 on cancer patients. The review identifies and discusses telehealth successes and failures, and the results provide a valuable guide to healthcare providers to better prepare their future operations. 

Following the COVID-19 pandemic, the importance of disease screening using smartphones has been stressed further. The final article [8], by Moses et al., involves a systematic review of the literature to examine the use of mobile apps for disease screening and technology acceptance among the users and healthcare practitioners. The results could inform future research on assessing mobile apps as a reliable screening tool.

## 3. Conclusions

This Special Issue was created to collate original research papers and review articles that explore new techniques, solutions and applications in e-health and mobile health monitoring. The collection of the eight articles in this Special Issue demonstrated the variety of studies in this domain and a rich repertoire of approaches and technical solutions identified by the authors in order to address the complex needs of healthcare stakeholders, particularly in the Australian context. The entire collection of eight articles makes an important contribution to the field of digital health in general, and technological advancements for health monitoring in particular. This said, the opportunities do not come without challenges and we would like to thank the authors for explicitly describing the special conditions for making their propositions successful, as well as highlighting the potential real-life challenges for implementing such solutions, especially based on the systematic reviews they conducted of the areas of telehealth, mobile disease screening and smartphone sensing.

The articles and their results provide valuable information about potential challenges and opportunities for the research community in e-health and mobile monitoring, and identify some future research directions for those who are interested in conducting similar research in the future.
